# Effects of Social Media Usage on Consumers’ Purchase Intention in Social Commerce: A Cross-Cultural Empirical Analysis

**DOI:** 10.3389/fpsyg.2022.837752

**Published:** 2022-05-11

**Authors:** Shangui Hu, Zhen Zhu

**Affiliations:** ^1^College of Business Administration, Ningbo University of Finance and Economics, Ningbo, China; ^2^School of Economics and Management, China University of Geosciences, Wuhan, China

**Keywords:** social media usage, purchase intention, cultural intelligence, cultural distance, social commerce, cross-cultural background

## Abstract

Social commerce has produced enormous economic benefits as well as challenges for organizations, individuals, and industries. However, social media usage does not necessarily generate users’ intention to purchase on social commerce websites. How social media usage influences users’ purchase intention on social commerce websites still deserves more scholarly attention and this seems particularly important when social commerce transcends borders and countries. Taking an interdisciplinary perspective, the current study adopted a survey research method and identified the roles of social media usage in arousing users’ purchase intention on social commerce websites in a culturally diversified environment. The data was collected from 2,058 international students coming from 135 countries and was analyzed using MPLUS based structural equation modeling. The research unveils the pathway whereby social media usage serves to generate users’ purchase intention on social commerce websites. Importantly, users’ cultural intelligence has been found to play a significant role mediating the effects of social media usage on users’ intention. Further, cultural distance was found to attenuate the effects of social media usage on cultural intelligence. Based on the research findings, the study suggests that social commerce practitioners should be fully aware of the enabling roles of social media and cultural intelligence as well as the deterring role of cultural distance when arousing customers’ purchasing intention in cross-cultural business operations. Any measures facilitated by social media usage to improve international consumers’ cultural intelligence and mitigate the negative effects of cultural distance are supposed to be effective to enhance their purchasing intention. Accordingly, the study confirms the mutually melt and integrative relationships between information technology advancement and business prosperity in cross-cultural environment, which eventually contribute to sustainable development of society.

## Introduction

The advancements of Web 2.0 technologies and the burgeoning applications of social media have dramatically revolutionized the performing effectiveness of business structures, models and operations (Huang and Benyoucef, 2015; Busalim and Hussin, 2016; Hariguna and Rachmawati, 2019; Zheng et al., 2020; Algharabat and Rana, 2021; Adiandari, 2022). One notable evolution is the emerging phenomenon of social commerce, which profoundly changes the business operations from vendor-push models into a user-driven environment (Wigand et al., 2008; Curty and Zhang, 2013; Lu et al., 2016; Alnoor et al., 2022). As a new emergence of business operations, social commerce allows consumers to interact actively with their peers to share commercial-related information and influence other consumers’ online purchase behaviors (Lin et al., 2017).

Although social commerce has achieved tremendous success since it was coined as a new form of business practices, it is reported that almost half of social media users showed no purchase intention on social commerce websites (Ko, 2018). More evidences have been found that people are not necessarily influenced by their peers using social media for online shopping behaviors because of potential negative effects including poor customer service and websites quality (Gutama et al., 2021), inaccurate information, privacy disclosure and fraud on consumers (Lickel et al., 2000; Mikalef et al., 2017; Paireekreng et al., 2019; Wang and Herrando, 2019; Chawla and Kumar, 2021). In other words, users’ purchase intention is not equally influenced by their connections with other users shopping online, and the behavior of using social media does not necessarily increase the likelihood of users’ purchase on social commerce websites (Kim and Park, 2013). As such, it is of great significance to examine the mechanism whereby individuals’ usage of social media generates their purchase intention on social commerce sites.

To keep up with the globalization pace of the 21st century, successful business relies heavily on international market and world-wide business operations (Chen et al., 2010). Accordingly, social commerce has integrated into the world economy as an important component of business operations in multinational companies (MNCs) (Friedman, 2006). Take alibabagroup.com as an example, the company offers millions of products to international consumers from over 190 countries each year (Deng and Wang, 2016). This suggests that social commerce transcends national borders and rates itself as an alternative option for both local and international consumers (Silbiger et al., 2021). However, cultural features, which to a large extent differentiate one country from another, vary in countries no matter how closely those countries are connected (Frijns and Garel, 2021). In this regard, cultural differences between countries create a more complex international business environment for social commerce operation. Albeit that few studies have identified the effects of culture on customer’s perceived benefits, word-of-mouth, service quality perceptions and business relationship quality (Schumann et al., 2010; Hoppner et al., 2015; Tang, 2017), how cultural differences influence consumers’ engagement in social commerce needs more empirical studies. As previous research indicated, increasing consumers’ cognitive, motivational and behavioral capabilities to deal with cross-cultural issues is an efficacious way to mitigate the negative consequences brought by cultural differences on their psychological and cognitive changes (Hu et al., 2020). However, there is paucity of literature explicating the role of cultural differences played in the cross-cultural business environment. In light of the globalized economy, social commerce has demonstrated its power to facilitate the rapid growth of cross-border commerce. As dominating features distinguishing one country from another, cultural differences deserve more scholarly efforts. In particular, there is lack of a complete picture about how cultural differences affect the process of developing consumers’ emotional and cognitive capability by leveraging on social media, thereby influencing their purchase intention when they surf social commerce websites in an environment with foreign cultural novelty. Therefore, the current study endeavors to address the above mentioned research gaps by answering the following questions:

Question 1: *In social commerce operations, how different dimensions of social media usage affect consumer’s purchase intention, particularly in cross-cultural business settings?*

Question 2: *How is the functioning of social media usage on consumer’s purchase intention influenced by cultural differences?*

In order to address the above research questions, we adopted the perspective of the social learning theory (Bandura, 1978). Scholars argue that the learning approach changes consumers’ cognitive and affective appraisal first before influencing their purchase intention (Chen et al., 2017; Friedrich et al., 2019). More specifically, we contend that social media usage, whether for seeking information or socializing, improves users’ cognitive and emotional aspects through interaction among community members and information exchange in social networks (Huang and Benyoucef, 2013; Hu et al., 2021a). The learning behaviors contribute to consumers’ knowledge and understandings of foreign cultures embedded in relevant products or conveyed through social commerce websites. During the process of users’ interaction with the environment and interplay between cognitive and emotional functions during the learning process, social media usage changes their attitudes toward the products and social commerce websites, and eventually influences their purchase-making intention and their final behaviors (Lorenzo et al., 2012).

This research makes several important contributions to the extant literature about social commerce in cross-cultural business environment. First, whereas loads of research work has focused on the functionalities of social media usage and antecedents determining consumers’ purchase intention (Fu et al., 2020; Jin et al., 2022), this study enriches the extant literature by examining the respective effects exerted by two dimensions of social media usage on consumers’ purchase intention in social commerce operations. Second, the majority of relevant studies have examined the direct effects of website features of social media platforms on consumers’ purchase intention. The study explicates the underlying mechanism whereby social media usage influences consumers’ purchase intention in social commerce environment. Third, in terms of the complexity of different cultural values, this study further examines how the extent of cultural differences acts as a noisy channel to affect the functioning of social media usage on potential consumers’ cultural intelligence, thereby influencing consumers’ purchase intention on social commerce websites. Fourth, the study expands the extant research on social commerce into far more complicated cross-cultural settings. Given that cross-cultural business environment is characterized with more unexpected risks and challenges than a singular cultural context, the study explicates the significant role of cultural intelligence invigorating the success of social commerce in cross-cultural business backgrounds.

**FIGURE 1:**
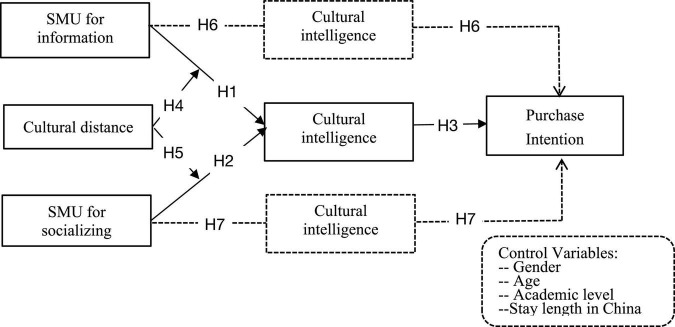
Proposed research model. The dotted line refers to two mediation effects of cultural intelligence.

The remainder of the paper contains the following parts. The second section is about the literature review, which is followed by hypotheses development. Thereafter, we presented the research methodology and data analysis. Based on data analysis, discussion part was presented. After that, research limitations and avenues for future efforts are discussed. And the conclusion part ends the whole paper. The proposed research model was depicted in Figure 1.

## Literature Review and Proposed Hypotheses

### Social Commerce and Social Learning Theory

Defined as “exchange-related activities that occur in, or are influenced by, an individual’s social network in computer-mediated social environments, where the activities correspond to the need recognition, pre-purchase, purchase, and post-purchase stages of a focal exchange” (Yadav et al., 2013, p. 312), the model of social commerce has been created as a result of the advancement of information technology and communication tools (Hajli, 2015; Sun et al., 2019; Nacar and Ozdemir, 2022). As a new emergence of business operation model, social commerce enables vendors to interact with consumers through social media platforms and enables consumers themselves to communicate and exchange information about vendors’ offered services and products (Aral et al., 2013; Friedrich et al., 2019). Three major attributes have been identified for social commerce operations, namely information technologies, social interactions, and organized commercial activities (Liang and Turban, 2011; Wang C. et al., 2019). Mediated by social media, social commerce involves the convergence of both offline and online environments (Zhou et al., 2013; Jami Pour et al., 2022). Two distinct categories of social commerce are summarized according to different business practice modes: (a) commercial activities that happen in traditional e-commerce websites where social communication technologies have been added to facilitate interaction between consumers themselves and between retailers and consumers; (b) commercial activities that happen in social networking sites where commercial features including transactions and advertisements have been incorporated (Zhang and Benyoucef, 2016; Chen et al., 2017).

To meet up with the demands of a globalized economy, multinational companies cross industrial and national borders to seek, access, and acquire new avenues for benefits attainment (Gassmann et al., 2010). In recognition of the conveniences and low costs yielded by applications of social media, business practitioners start their investment to development the platforms where social commerce is grounded. In such cases, social commerce serves as an innovative channel to facilitate firms to navigate international markets and access both local and international consumers through internet connections (Kotabe et al., 2002). Although the social commerce is burgeoning worldwide, yet there are still tough encounters that impede consumers’ purchase intention and shopping behaviors (Van Heel et al., 2011; Sun et al., 2019; Algharabat and Rana, 2021). Previous research has identified that e-commerce conducted internationally has to deal with more complex cross-cultural settings where cultural differences between countries exist as dominating stressors hampering consumers’ online experiences (Hu et al., 2018, 2020). In this aspect, more studies conducted with empirical methods are complementary measures to unveil how social commerce consumers’ intention to purchase is aroused in more complex settings featured by cultural novelties and uncertainties.

From the perspective of social media users, when they browse social media networks to search for relevant products’ information and interact with others for exchanging ideas, they are in actuality learning from others before they develop their purchase intention (Huang and Benyoucef, 2013). From the perspective of social learning theory (SLT), all learning is underpinned with two fundamental assumptions. The first one is about the external interaction between learners and their exposed social, cultural, and material environments, and the second refers to the internal psychological process of knowledge and skills acquisition and explication based on previous learning. Only when the two processes are involved together, not separately, can learning behaviors take place. In parallel with two underlying processes through which learning takes place, three dimensions of learning have been depicted, namely cognitive dimension, emotional dimension, and social dimension (Illeris, 2003; Chen et al., 2017). The cognitive dimension is covered in the internal process and deals with the learning content about knowledge and skills which builds up learner’s cognitive framework and their ability. Likewise, the emotional or psychodynamic dimension is included in the internal process and encompasses necessary mental energy, feelings, and motivations for completing the process. The social dimension of learning depicts the external interaction between learners and their environment including their participation, communication, cooperation, and even integration in communities and society (Illeris, 2003). It is noteworthy that the three dimensions of learning are interdependent and embedded in the social, cultural, and material environments, and the psychological process is crucially dependent on the external interactions. Since its initiation, SLT has demonstrated its explanatory power to explicate human’s development and behaviors, and has been adopted to elaborate on customers’ social commerce behaviors (Chavis, 2011; Hu et al., 2018).

The current research adopts the social learning theory as the theoretical lens because the theory offers a structured approach as a response to explicate an individual’s psychological changes and behavioral patterns in different social and cultural contexts (Illeris, 2003; Chen et al., 2017). Albeit social commerce demonstrates itself as a special form of e-commerce, the fundamental social psychology backing up consumers’ online shopping intention or behaviors still lies in social learning (Chen et al., 2017). SLT indicates that consumers’ purchase-making decision or intention is determined both by an external interaction between consumers and their exposed environments, and by an internal psychological process to acquire and elaborate environmental elements. In a cross-cultural environment, social media platforms provide conditions for consumers to learn about foreign cultural knowledge posted on the websites or embedded in offered products (Hu et al., 2021a). Further, consumers also learn from observations of others’ social commerce experiences by means of information exchange and interaction with community members (Hu et al., 2017). During the external interaction with environmental factors, social media users experience cognitive, emotional, and behavioral changes which were originally interpreted as their development of the capacity to manage cross-cultural encounters, namely cultural intelligence (Ang et al., 2007; Hu et al., 2018). According to SLT, those changes may finally arouse users’ purchase-making decision or intention as a result of an integrated interplay between the functions of cognition and emotion during the process of learning.

### Social Media Usage (Informational and Socializing) and Cultural Intelligence

Due to the advancement of information technology, social media has permeated into every territory of contemporary society and exerts vast impacts on individuals’ and organizations’ survival and success by functioning as a platform for individuals’ social interaction and information exchanging (Kauffman et al., 2017; Mohammed and Qhal, 2020; Lin et al., 2021). Strong evidence indicates that individuals are driven by utilitarian and hedonic value orientations when they use social media platforms (Wang, 2010), and generally gratified with informational and socializing purposes (Hu et al., 2020). More specifically, social media usage for informational purpose is to gratify individual cognitive needs by searching for information to solve confronted problems, whilst social media usage with socializing motivation is to satisfy individual emotional needs by establishing and maintaining interpersonal relationships through networking sites (LaRose, 2009; Hu et al., 2021b; Guan et al., 2022).

When accommodated in a culturally novel environment filled with cultural conflicts and potential risks, individuals are supposed to manage cultural conflicts and unexpected situations (Ward et al., 2011), which may trigger individuals’ negative emotions such as loneliness, depression, and psychological disorder (Hu et al., 2020). Social media provides opportunities for individuals to search for desired information and establish and maintain social networks (Hughes et al., 2012; Khaola et al., 2022). In this vein, social media is closely associated with individuals’ international exposure by serving to develop their cultural intelligence and facilitate their working and living conveniences including online shopping experiences.

Coined by Earley and Ang (2003), the conceptualization of cultural intelligence (CQ) represents people’s capacities to manage cross-cultural issues in a foreign culturally novel environment. Since its initiation, CQ is regarded as a key capacity enabling international expatriates to adjust themselves to a culturally new land, develop personal abilities and conduct successful overseas businesses (Imai and Gelfand, 2010; Chen et al., 2012; Kim and Dyne, 2012). Numerous studies have identified that individuals with a higher level of CQ demonstrate more desirable outcomes, such as leadership performance (Guang and Charoensukmongkol, 2022), self-efficacy (Hu et al., 2018; AlMazrouei and Zacca, 2021), cross-cultural adjustment (Hu et al., 2020), organizational embeddedness and knowledge sharing (Stoermer et al., 2021), and global leadership (Ang and Van Dyne, 2015). On the other hand, individuals with a lower level of CQ may experience unexpected problems in their learning and working environments, such as discrimination, loneliness and depression in cross-cultural settings (Baltacı, 2017; Karatas and Arpaci, 2021). In light of the significance of CQ, scholars endeavored a lot to explore the antecedents influencing the development of individuals’ CQ. Studies have found that both environmental elements and personal attributes affect the development of individuals’ CQ, such as multicultural experiences and training programs (Hu et al., 2017), information technology (Hu et al., 2018), self-efficacy (Hu et al., 2018) and personality dimensions (Presbitero, 2018; Hu et al., 2020). Albeit research has confirmed that CQ functions as a mediating mechanism (Hu et al., 2020), how CQ mediates the relationships between dimensions of social media usage and customers’ purchase intention in cross-cultural social commerce environment still lacks sufficient empirical evidence.

Regarded as being multidimensional, cultural intelligence comprises four dimensions which cover metacognitive, cognitive, motivational, and behavioral aspects of CQ (Earley and Ang, 2003; Stoermer et al., 2021). As a fundamental element, metacognitive CQ is defined as an individual’s cognitive power to control consciousness to acquire and comprehend knowledge about a foreign culture and make necessary mindset adjustment to expected and unexpected foreign situations (Ang et al., 2007; Hu et al., 2018). Cognitive dimension of CQ pertains to the systematized knowledge learned about the foreign culture individuals are exposed to, which includes legislated norms, underlying values, long-term traditions and social practices (Triandis, 2006). With good mastering of this kind of intelligence, individuals are able to master know-how to distinguish cultural differences existing among different cultures (Hu et al., 2020). Likewise, motivational dimension of cultural intelligence demonstrates an individual’s sufficient motivations to cope with foreign cultural related issues successfully (Hu et al., 2021b). Accordingly, behavioral CQ represents an individual’s behavioral ability to respond appropriately to expected and unexpected encounters in a culturally diversified environment (Hu et al., 2018; Stoermer et al., 2021), which contributes to individuals’ general adjustment, working performance and learning results in a cross-cultural environment.

According to SLT, learning happens through external interaction processes and internal psychological process with cognitive and emotional changes. Firstly, when situated in a culturally diversified business environment, social media users are inclined to use social media to acquire related information for capture and comprehension of foreign cultural knowledge that are originally different from that of their home countries (Hu et al., 2018). Further, SLT suggests that the observation of others’ behaviors and experiences is also a basic learning approach (Chen et al., 2017). Users observe others’ shopping experiences by reading their posted information on social media and learn about their shopping experiences and comments shared on the social commerce websites (Bugshan and Attar, 2020; Stoermer et al., 2021). Such experiences facilitate users to expand their knowledge infrastructure and self-consciousness with attained values, conventions, values, and cultural practices in international business settings, which contributes to the development of individuals’ both metacognitive and cognitive dimensions of CQ. In addition, established metacognitive CQ and cognitive CQ facilitate individuals with sufficient interest and motivation to be engaged in complicated cross-cultural issues. Moreover, more acquaintance with foreign cultures embedded in the products and social commerce websites arouses individuals to have more interests and motivations to manage effectively their behaviors and decision-making patterns, which contributes to their development of individuals’ motivational CQ. Accordingly, equipped with orientated self-consciousness, sufficient cultural knowledge, and strong intrinsic motivation, individuals are supposed to behave appropriately in a culturally diversified social commerce environment (Glass and Westmont, 2014; Guang and Charoensukmongkol, 2022). Based on the above statements, we make the hypothesis as follows:

**H1**: Social media usage for informational purpose has positive associations with cultural intelligence.

Social learning theory underlines that personal development and psychological changes of human beings are triggered by the interaction effects between environment’s external elements and individual’s inner psychological changes (Bandura, 1978). Within the theoretical framework of SLT, social media can be acknowledged well as an environmental factor to interact externally with learners (Illeris, 2003; Hossain et al., 2021). As a social dimension of SLT, learners are actively involved in establishing and maintaining interpersonal relationships with community members on social media websites (Weller, 2016; Matsuda, 2022). In particular, international users tend to be more engaged in social media networking when they are exposed to novel culture and are confronted with numerous uncertainties and risks (Zhang and Goodson, 2011; Karatas and Arpaci, 2021). As such, they utilize social media to socialize with people who are culturally different (Hu et al., 2018). During the interaction process, social media users leverage on online opportunities to exchange necessary information related to cross-cultural issues with their peers, seniors, and colleagues from diversified cultural backgrounds. Moreover, the attained information from online interaction seems much more reliable, accurate, and trustworthy than that sought directly from business websites (Hur et al., 2017; Soleimani, 2021). Therefore, social media usage offers smart channels for potential consumers to interact with others from various cultural backgrounds for information, knowledge sharing and idea exchange purposes (Ghahtarani et al., 2020; Jen and Lin, 2021). This conduit increases their knowledge infrastructure and boosts their consciousness and motivations to learn more about another foreign culture (AlMazrouei and Zacca, 2021). Additionally, social media usage contributes to the development of maintaining extant interpersonal connections and establishing new social connections as well, which enables users to develop appropriate behavior capabilities in a culturally novel environment (Bonebrake, 2002; Hu et al., 2020; Rakhmansyah et al., 2022). Based on the above statement, we can logically hypothesize that:

**H2**: Social media usage for socializing purpose has positive associations with cultural intelligence.

### Cultural Intelligence and Purchase Intention

In an online business environment, one cause of customer uncertainty is asymmetric information overloaded on users’ decision making (Hwang et al., 2014). Users with lower levels of CQ are easy to be harnessed by overloaded information and fail to differentiate useful information from the useless (Hu et al., 2018; Yazdanshenas, 2021). In contrast, higher levels of CQ facilitates users to sort out the useful from a chaotic reservoir of information, enabled by acquired knowledge about the foreign culture, tradition, values embedded in products and business environments (Hu et al., 2020). In this regard, CQ enables social media users to leverage on useful information both from social media and comments made by other consumers, thereby facilitating to develop their purchase intention on social commerce websites.

Empirical studies indicated that individuals with CQ demonstrate to be more culturally engaged with locals and more acceptable than individuals with relatively lower level of CQ (Earley and Ang, 2003; Imai and Gelfand, 2010). This can be interpreted in the way that individuals with CQ have more cultural consciousness and cognitive knowledge to develop and maintain trustworthy interpersonal relationships by interacting with those in the online community with attributes of their intrinsic motivation and behavior capability (Ng, 2013). In particular, sufficient communications facilitate users to establish trust-based integration into the online community, and allow users to understand, predict, and meet the group-normative expectations (Ng, 2013; Guillaume et al., 2014; Prabowo et al., 2021). Further, research has demonstrated that trust is regarded as a basic psychological element to motivate social media users to surf social websites and demonstrate other online behaviors in social commerce environment (Stewart, 2003; Liang and Turban, 2011; Leong et al., 2020). This proposition supports the notion that well-established social relationships with online community members relieve users’ concerns of being defrauded by online uncertainties and risky events, and thus drives them to develop purchase intention on social websites more possibly (Wang X. et al., 2019).

Thus it can be logically hypothesized that:

**H3**: Cultural intelligence has positive associations with users’ purchase intention.

### The Moderating Effects of Cultural Distance

As a fundamental element distinguishing one group from the other groups, culture is regarded as a contextual variable which influences individuals’ motivation, attitudes and behaviors (Chen et al., 2018). In the competitive global market, culture is confirmed to repeatedly affect consumer’s cognitive patterns and behaviors (McCort and Malhotra, 1993; Schumann et al., 2010). Cultural distance, interpreted as the extent of cultural differences distinguishing host country from individual’s home country (Shenkar, 2001; Silbiger et al., 2021), describes the disparities existing between countries in terms of development level, education, language, business, legislated systems, conventions, cultural values, and connections level (Froese and Peltokorpi, 2011; Liu et al., 2021).

Since its evolution from physical distance, cultural distance has received burgeoning attention from scholars engaged in the literature of cross-cultural studies and international business. Accordingly, the conceptualization has been examined as an influential factor to test the robust effects of culture on international business patterns, trade flows, market entry, products’ design philosophy and joint-venture performance (Froese and Peltokorpi, 2011; Silbiger et al., 2021). As one of the dominating stressors perceived by individuals in a culturally different community, cultural distance has been interpreted from the perspectives of cultural novelty and toughness (Mendenhall and Oddou, 1985; Black et al., 1991), which exerts effects on individuals’ cross-cultural performance and psychological stability (Chen et al., 2010). However, there is a paucity of literature researching its effects on social commerce. Based on the social learning theory, scholars proposed that cultural distance is most likely to play a boundary conditional role influencing individual-level perceptions of stressors and stability of personal attributes, and thus brings negative effects to personal development, because it captures the most complicated aspects and challenges inherent in culturally diversified contexts (Chen et al., 2010).

When a high cultural distance between countries is perceived, it indicates that users have more inherent obstacles to comprehend the information when they use social media for informational purposes. In this regard, individuals feel more difficult to understand and accept cultural nuances and concepts embedded in products and social commerce websites. For the purposes of knowledge access and acquisition, when exposed to a foreign country with more cultural distance, users are supposed to allocate more personal resources to develop cultural intelligence by searching relevant information from social media. More specifically, when exposed to a culturally distant environment, users feel more inherent difficulties to search for, comprehend, and absorb appropriate information from social media for personal development. This is interpreted by the resource allocation model which illustrates that individuals are inhibited by lack of knowledge to manage assigned tasks despite of enormous efforts made (Yeo and Neal, 2004; Liu et al., 2021). Thus cultural distance is more likely to undermine the effects exerted by informational social media usage on individual’s cultural intelligence.

**H4**: The effects of social media usage for information on individual’s cultural intelligence are attenuated by cultural distance.

Likewise, an environment with higher cultural distance becomes more detrimental for users to socialize with others than the environment with lower cultural distance (Tett and Burnett, 2003; English et al., 2021). Previous studies have examined that cultural distance strongly impacts social relationships and personal communications in cross-cultural contexts (Galchenko and van de Vijver, 2007). Scholars researching conflict management and cross-cultural studies consider that people from different cultures may find it more challenging to interact with each other (Ma, 2010). Accordingly, when users use social media websites with a socializing purpose to establish or maintain interpersonal relationships, they are deeply influenced by the homophily, which refers to the degree of cultural distance (Hu et al., 2020). A culturally distant business environment hinders users to adopt an effective strategy to communicate with those who have different conceptual understandings about culture and culturally rooted products, which finally minimize the effectiveness of using social media to socialize with others for developing individuals’ cultural intelligence (Silbiger et al., 2021). Further, cultural distance impedes users from using social media websites to integrate into the social commerce community. For example, Chinese culture is relatively distant for users who are from western countries, because China is a typically eastern country which is deeply rooted in distinct collectivism culture, while western countries unanimously keep the value of individualism culture. As such, western consumers may fail to capture the integration of traditional cultural features reflected into Chinese products and business promotion strategies (Chen and Peng, 2008; Liu et al., 2021). Thus obstacles generated by cultural distance may result in failure of communication efforts and idea exchanges during the process of individuals’ interacting with the local people through social media. In this regard, cultural distance separates users to be fully immersed into the online community. This also indicates that cultural distance prevents users from developing cultural intelligence when they use social media with the purpose of socializing with others in culturally novel settings. Thus it can be logically hypothesized that:

**H5**: The effects of social media usage for socializing on individual’s cultural intelligence are attenuated by cultural distance.

### The Mediating Effects of Cultural Intelligence

Scholars argue that social commerce is a multidimensional concept in actuality and consumers expected benefits from using social commerce come from one of three aspects or all of them, namely utility benefits, social benefits, and hedonic benefits (Osatuyi et al., 2020). Further, it has been identified that relationships between antecedents and users’ intention to use social commerce could be non-linear, not direct, due to environmental and users’ psychological factors’ changes (Lankton et al., 2016; Osatuyi et al., 2020). As mentioned above, in cross-cultural contexts, the relationships between customers’ social media usage and their purchase intention in social commerce deems to be more complicated in light of environmental complexities (Hu et al., 2018). In this regard, customers’ potential interest to use social commerce relies heavily on seeking information and socializing with platform members they are familiar with or not through social media usage. As a result, potential customers’ leveraging on social media for utilitarian and hedonic purposes result in changes of their cultural intelligence by increasing cognitive, motivational and behavioral capabilities. And those improved capabilities mobilize themselves to have better understandings about social commerce, thereby increasing their intention to use social commerce.

Since its creation, cultural intelligence has attracted scholars’ attention due to its explanation power to elicit individuals’ capabilities to deal with cross-cultural related issues (Ang et al., 2007; Pidduck et al., 2022). It is worthy to note that increasing research has uncovered that cultural intelligence plays as a key antecedent to predict individuals’ positive psychological changes and cross-cultural behaviors (Ang and Van Dyne, 2015; Hu et al., 2020, 2021a). More importantly, cultural intelligence has been identified as a mechanism to explicate how and why individuals take actions and perform effectively in complicated cross-cultural environment (Lee and Sukoco, 2010; Hu et al., 2017; English et al., 2021). Accordingly, we contend that cultural intelligence acts as a mechanism as well when users utilize social media platforms to increase their purchase intention in social commerce by developing cultural intelligence as a prerequisite to better capture the content, design, delivered information and word-of-mouth about the products originated from different cultures. Numerous studies have also elicited that cultural intelligence plays the mediating roles pertaining to the final cross-cultural performances (Lee and Sukoco, 2010; MacNab and Worthley, 2012; Hu et al., 2021a).

Based on above statements, we hypothesize that:

**H6**: Cultural intelligence mediates the relationship between social media usage for information and purchase intention.
**H7**: Cultural intelligence mediates the relationship between social media usage for socializing and purchase intention.

## Methodology

The study employed both Spss23 and Mplus8 statistical software to analyze the data. Descriptive statistics, means, variances and correlations of variables were calculated through Spss23. In addition, considering the advantages of Mplus8, this study carried out regression analysis by constructing structural equation models using Mplus8. Up to date, Mplus8 is regarded as one of the most popular and powerful software available for latent variable analysis. Firstly, Mplus8 can handle not only categorical latent variables but also continuous latent variable models as well as complex models with both variables. Moreover, it is specially designed for the analysis of various latent variable models, for missing values and for complex survey data. Secondly, Mplus8 can also analyze cross-sectional and longitudinal data, hierarchical data (multilevel analysis) and data from different aggregates (multi-group analysis). Finally, Mplus8 also offers a graphical manipulation function that allows the software to automatically help program and produce the corresponding results through graphical manipulation.

### Sample and Procedure

To further examine the above proposed hypotheses, a survey was designed to measure the variables contained in the proposed research model. With generous assistance from more than 40 public universities offering international education in China, the questionnaires were delivered to international students who were studying their degree or non-degree programs at those higher institutions. International students studying in China were chosen for the current research samples mainly for three reasons. First, international students are a special group of international consumers. Application of social media for information and social interactions has become a ubiquitous phenomenon among them. However, this special group of consumers is rarely investigated by scholars of social commerce research. Second, international students share similarities a lot with other international consumers confronted with unexpected risks and uncertainties in a multicultural environment (Hu et al., 2018). Third, most of the extant research was conducted in western cultural background. Meantime, China has made itself successfully be the first targeted country for international education in Asia with 492,185 international students registered in Ministry of Education of People’s Republic of China (2018). Examining how social commerce is conducted out of western cultures is a necessary supplementation to the literature of social commerce research.

Before the large scale delivery of the questionnaires, two pilot tests were conducted to ensure the validity of all variables. For the first pilot test, 40 international students were invited to offer their responses to variable items. And 3 scholars engaged in relevant research fields were invited to assess the students’ responses and variables’ validity and make necessary revisions to the wording and paraphrases. Further, interviews were conducted among the first group pilot students to revise those items in order to ensure all those items to match their real *status quo* in China. To guarantee all revisions appropriate, a second pilot test was conducted among another group of 71 students after two weeks. The second pilot test demonstrated very good results. Afterward, the questionnaires were distributed through one of the largest survey platform Wenjuanxing^1^, which is more efficiently used by scholars to collect sensitive data than offline channels (Stewart and Bing, 2009). The website links were offered to all volunteer students with the help of university officials. During the 1-month process of collecting data, reminded information was sent to students to increase the response rate. And a final of 2058 questionnaires from 135 countries’ students were returned for data analysis. Among all participants, 1154 were male students and 904 were girls. Moreover, 65.89% students were from Asian countries and African students occupied 24.73%. Pertaining to their academic levels, more than 70% were bachelor or below bachelor degree holders. Further, almost half of all participants (46.65%) have been staying in China for more than 2 years. Table 1 shows the demographic statistics of the sample.

**TABLE 1:** Demographic statistics of the sample (*N* = 2058).

Category	Item	Frequency	Percentage (%)
Age	≤20 years old	365	17.74%
	21–25 years old	863	41.93%
	26–30 years old	473	22.98%
	≥31 years old	357	17.35%
Gender	Male	1154	56.07%
	Female	904	43.93%
Areas	Asia	1356	65.89%
	Africa	509	24.73%
	Europe	97	4.71%
	Oceania	17	0.83%
	America	75	3.64%
	Missing data	4	0.19%
Highest diploma	below bachelor	772	37.51%
	bachelor	681	33.09%
	master	493	23.96%
	doctor	112	5.44%
Stay length in China	Less than 6 months	446	21.67%
	6–12 months	127	6.17%
	1–2 years	525	25.51%
	More than 2 years	960	46.65%

### Measurements

Three international students were chosen for the pilot test, who were participating in degree programs and language training courses, to complete the questionnaires to help evaluate the measures we adopted from previous literature. Necessary revisions were made to the question wordings according to three students’ feedback to ensure the questionnaire items meet international students’ real status in China. In addition, three academic researchers in the relevant fields were invited to join in the work of reviewing the validity of all variables. All the measurement work was conducted with five-point scales from 1 to 5.1 represents users’ “strongly disagree” and 5 represents users’ “strongly agree.”

For the measurement of social media usage, six items were adopted from Hughes et al. (2012). 6 items were divided into two halves. The first three items were used to measure social media usage for informational motivation and the other half were used to measure social media usage for socializing motivation.

To measure the overall cultural intelligence, we used 20 items, which were developed originally by Ang et al. (2007). To measure the metacognitive, cognitive, motivational and behavioral aspects, 4, 6, 5 and 5 items were used respectively.

Further, we measured users’ purchase intention on social commerce websites by adopting three items proposed by Shin (2013). To measure the cultural distance, we used 6 items developed by Chen et al. (2010), to measure the extent of cultural differences between China and students’ home countries. Sample items included “How similar are religions and rituals in China similar to your own country?” and “How similar are values in China similar to your own country?” Measures were assessed ranging from 1 for “very similar” to 5 for “not similar at all.”

We also controlled for participants’ age, gender, length of stay in China, and their education level to avoid any possible confounding effects identified in previous literature (Hu et al., 2020).

## Hypotheses Analysis

### Constructs Validity

Preliminary analysis was performed to test potential common method bias, constructs’ reliability and validity. The statistics shown in Tables 2, 3 show that the loadings for all standardized factors ranged from 0.747 to 0.915, which were above 0.6 criteria. The AVE values ranged from 0.688 to 0.908, greater than required 0.5 (Hair et al., 2020), and composite reliability was from 0.766 to 0.944, greater than required 0.7 (Bagozzi, 1981). All those statistics demonstrated the good convergent validity of all variables. Each variable’s indicated arithmetic square root of the AVE value was greater than the correlation between this and other variables (Fornell and Larcker, 1981). Thus discriminant validity was confirmed.

**TABLE 2:** Means, standard deviations, and correlation matrix (*N* = 2058).

Constructs	Means	SD	CQ	CD	SMUI	SMUS	PI
CQ	3.9223	0.65314	**0.753**				
CD	3.3768	0.90273	0.307**	**0.825**			
SMUI	3.8431	0.90465	0.389**	0.342**	**0.953**		
SMUS	3.8481	0.86333	0.435**	0.324**	0.641**	**0.830**	
PI	3.9342	0.80811	0.568**	0.282**	0.372**	0.413**	**0.910**

CQ, cultural intelligence; CD, cultural distance; SMUI, social media usage for information; SMUS, social media usage for socializing; PI, purchase intention; **p < 0.01 (two-tailed) and the diagonal is the arithmetic square root of the AVE value (bold values).

**TABLE 3:** Measurement of constructs.

Constructs	Dimensions	Loadings	CR	AVE	Cronbach’s alpha
SMUI	SMUI1	0.853	0.766	0.908	0.843
	SMUI2	0.887			
	SMUI3	0.885			
SMUS	SMUS1	0.825	0.869	0.690	0.763
	SMUS2	0.791			
	SMUS3	0.873			
meta-cognitive CQ	CQ1	0.894	0.944	0.808	0.912
	CQ2	0.897			
	CQ3	0.916			
	CQ4	0.888			
Cognitive CQ	CQ5	0.823	0.932	0.696	0.912
	CQ6	0.810			
	CQ7	0.841			
	CQ8	0.845			
	CQ9	0.847			
	CQ10	0.840			
Motivational CQ	CQ11	0.833	0.931	0.728	0.970
	CQ12	0.847			
	CQ13	0.874			
	CQ14	0.852			
	CQ15	0.860			
Behavioral CQ	CQ16	0.776	0.928	0.723	0.903
	CQ17	0.848			
	CQ18	0.884			
	CQ19	0.885			
	CQ20	0.885			
PI	PI1	0.905	0.938	0.829	0.897
	PI2	0.912			
	PI3	0.915			
CD	CD1	0.792	0.929	0.688	0.906
	CD2	0.812			
	CD3	0.866			
	CD4	0.866			
	CD5	0.886			
	CD6	0.747			

*CQ, cultural intelligence; CD, cultural distance; SMUI, social media usage for information; SMUS, social media usage for socializing; PI, purchase intention.*

Further, common method bias may exist when the response variations are the function of instrument rather than the real expressions of participants themselves. To assess the potential problem of common method bias, we used Harman’s one factor, suggested by Podsakoff et al. (2012). Five principal components were created (with an eigenvalue greater than 1). The first principal component explains less than half of the total variance, which indicates that the common method bias is not a serious problem in the current research.

### Hypothesis Testing

**FIGURE 2:**
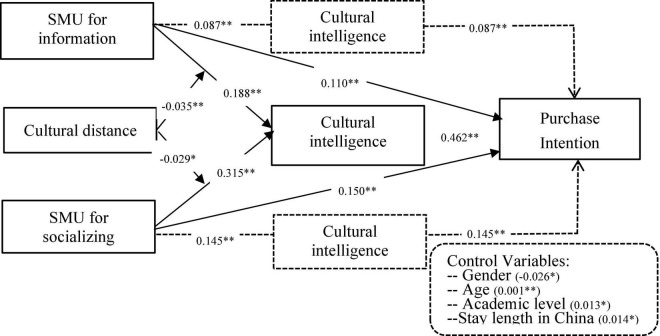
Results of the hypothesized model. SMU, social media usage. *p < 0.05 and ^**^p < 0.01.

In order to test the above proposed hypotheses, we conducted a regression analysis with Mplus8.3. As mentioned above, we also controlled for participants’ age, sex, educational level, and their stay length in China to avoid any potential confounding effects. The regression results were shown at Figure 2. Consistent with H1, informational usage of social media demonstrated positive relationship with cultural intelligence (*r* = 0.188, *p* < 0.01). Furthermore, socializing usage of social media demonstrated significantly positive relationship with cultural intelligence as well (*r* = 0.315, *p* < 0.01). Thus H2 was also supported. Thereafter, we also examined how cultural intelligence influences users’ purchase intention. The result indicates that cultural intelligence also had positive association with users’ purchase intention on social commerce websites (*r* = 0.462, *p* < 0.01). H3 was supported as well.

In addition, we examined the mediating functions of cultural intelligence in the proposed research model. A bootstrapping method was employed to test the mediation results. After 2000 iterations, cultural intelligence was found to mediate the relationships between two dimensions of social media usage and users’ purchase intention respectively (SMUI-CQ-PI, 0.085, 95% CI [0.046, 0.124]; SMUS-CQ-PI, 0.145, 95% CI [0.105, 0.187]). The mediating effects were both significant for the 95% confidence interval. And there is no zero between the lower and the upper. Thus H6 and H7 were both supported. (see Table 4).

**TABLE 4:** Results of the mediating effect.

Mediating effect	Estimate	SE	*P*	BC 99% CI	
				Lower	Upper
SMUI-CQ-ICS	0.087	0.015	0.000	0.046	0.124
SMUS-CQ-ICS	0.145	0.016	0.000	0.105	0.187

To examine the moderating effects of cultural distance, this study first decentralized the constructs of social media usage for information, social media usage for socializing and cultural distance. Following that step, we further created the interaction items between two dimensions of social media usage and cultural distance respectively and examined the effects of interactive constructs on cultural intelligence (Table 5). The result indicates that cultural distance attenuated the effects of informational social media usage on cultural intelligence (*r* = –0.035, *p* < 0.01). Thus H4 was supported. Further, cultural distance was also found to undermine the effects of socializing social media usage on cultural intelligence as well (*r* = –0.029, *p* < 0.05). Therefore H5 was supported as well.

**TABLE 5:** Results of hierarchical regression analyses for interaction.

Variable	Cultural intelligence				
	Model 1	Model 2	Model 3	Model 4	Model 5
Age	–0.021	0.001	0.001	0.001	0.015
Gender	–0.003	–0.042	–0.044	–0.033	–0.034
Stay length in China	0.002	0.012	0.013	0.006	0.008
Highest diploma	–0.015	0.014	0.018	0.006	0.009
SMUI		0.237**	0.229**		
SMUS				0.285**	0.278**
CD		0.142**	0.145**	0.135**	0.137**
SMUI*CD			–0.035**		
SMUS*CD					–0.029*
R^2^	0.001	0.189	0.192	0.221	0.223
ΔR^2^		0.189	0.003	0.221	0.002
ΔF		79.593**	7.274**	97.235**	4.949**

N = 2058. SMUI, social media usage for information; SMUS, social media usage for socializing; CD, cultural distance. Standardized regression coefficients are shown. *p < 0.05, **p < 0.01.

**FIGURE 3:**
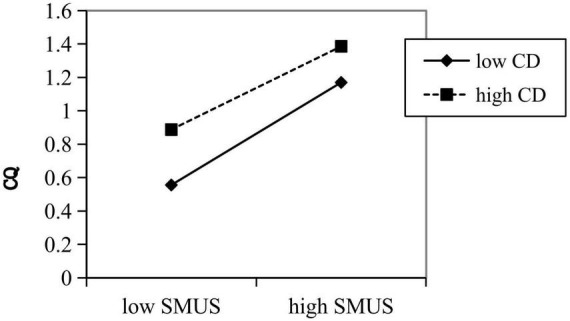
Moderating effect of cultural distance on the relationship between informational social media usage and cultural intelligence.

**FIGURE 4:**
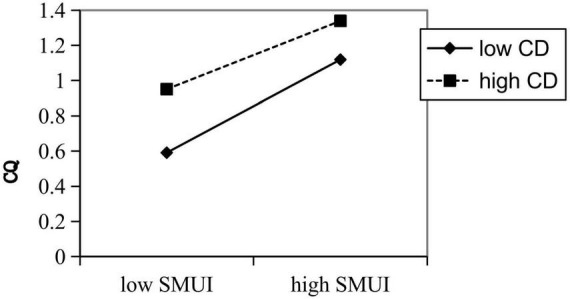
Moderating effect of cultural distance on the relationship between socializing social media usage and cultural intelligence.

The result of simple slope analysis further indicates that cultural distance mitigated the effects of social media usage, whether for information or socializing, on cultural intelligence (Figures 3, 4).

## Discussion

The research adopted an empirical method and examined the associations among social media usage, users’ purchase intention on social commerce websites, cultural distance and cultural intelligence. All proposed hypotheses were supported well after the regression analysis of the data collected from 2058 international students who came from 135 countries all over the world. The results indicate that two dimensions of social media usage, information and socializing, both significantly influence individuals’ purchase intention by increasing their cultural intelligence. However, cultural distance was found to significantly mitigate the positive effects of both informational and socializing social media usage on cultural intelligence.

### Findings

The research findings illustrated that the mechanism why and how social media usage exerts impacts on potential customers’ purchase intention in social commerce, particularly in complicated cross-cultural environment. More specifically, social media usage, whether it is for informational or socializing purpose, exerts positive effects on users’ purchase intention. The research findings partially keep in line with previous ones to some extent, which suggested that social media usage influences users’ online psychological well-being and online intention (Ellison et al., 2007; Hu et al., 2017, 2018; Priyo et al., 2019; Wang et al., 2021).

Further, the research unveiled how purchase intention in social commerce takes place in cross-cultural environment. The study has identified that when individuals are exposed to culturally diversified environment, social media usage helps them to develop their cultural intelligence, thereby enhancing users’ purchase intention. Further, cultural intelligence was found to partially mediate the relationships between two dimensions of social media usage and users’ purchase intention. The research findings keep compatible with prior research claiming that cultural intelligence plays as a conduit whereby social media usage functions on users’ motivation, attitudes and behaviors, particularly in a culturally diversified business environment (Hu et al., 2017). In addition, the research discovered the role of cultural distance attenuating the effects exerted by both two dimensions of social media usage on cultural intelligence. The result indicates that cultural distance undermines the positive influences of social media usage on users’ cultural intelligence. In particular, cultural distance exerts more negative effects on social media usage for informational purpose than for socializing purpose. This might be explained that socializing social media usage has been regarded as an efficacious way to facilitate establishment of interpersonal trust and mutual understandings, which may contribute to diluting the negative effects emanating from cultural distance (Lu et al., 2016). As such, the current research has confirmed that cultural distance impairs the functionalities of social media usage to develop individual’s cultural intelligence in the environment with cultural novelty, thereby influencing users’ purchase intention from their surfed social commerce websites. The research findings also match well with previous literature stating that cultural distance is a boundary element deterring an individual’s psychological well-being and personal development in cross-cultural settings (Froese and Peltokorpi, 2011; Ng, 2013).

### Research Implications

Based on social learning theory, an interesting research model was proposed for the current cross-cultural social commerce study. Accordingly, research hypotheses were tested with empirical evidence. The research findings make important contributions to the extant literature researching social commerce in several aspects.

First of all, the study partially unveils the underlying mechanism of social commerce which is supposed to be conducted in the environment transcending borders with cultural novelty. As for as we know, this effort is among the first trials investigating the functioning role of social media usage on users’ purchase intention, particularly with a cross-cultural background for social commerce operation. Although tremendous efforts have been made to uncover the determinants influencing users’ online intention for social commerce behaviors (Stewart, 2003; Liang and Turban, 2011; Chen and Shen, 2015), there is paucity of efforts endeavored to examine how two aspects of social media usage correlate with users’ purchase intention for their social commerce real practices. The research findings confirm that two aspects of social media usage facilitate users’ purchase intention on social commerce websites, which advances our understandings of social media and extends social commerce research to a much more complex cross-cultural environment. Undoubtedly, the research supplements the nomological network of the extant social commerce research.

Secondly, the current research also integrates cross-cultural studies into the social commerce research field by identifying that cultural intelligence plays different roles mediating the effects of two different dimensions of social media usage respectively on users’ purchase intention in social commerce practices. Although researchers have identified the significant role of cultural intelligence enhancing expatriates’ cross-cultural adaptation, personal development (Li and Tsai, 2015; Hu et al., 2020), few previous studies have identified how individual’s cultural intelligence functions in social commerce operations. The research findings confirm the important role of cultural intelligence mediating the effects of social media usage on users’ purchase intention as an effective conduit. It is worthy to note that cultural intelligence functions differently when mediating the relationships between two dimensions of social media usage and users purchase intention respectively. The research findings shed light on the different mechanisms whereby social media usage influences users’ purchase intention in culturally diversified social commerce environment. The findings keep compatible with previous studies revealing the different mediating roles of cultural intelligence in a novel environment (Hu et al., 2017, 2021a). In addition, the current research is also among the first trials investigating how one contextual factor confounds another contextual factor to function on social commerce. To be more specific, cultural distance was found to act as a boundary condition to prevent social media usage from developing users’ cultural intelligence in a culturally diversified social commerce environment. More specifically, social media usage demonstrates more positive associations with expatriates’ cultural intelligence in a less culturally distant environment where the environment is less complex due to relatively enjoyable cultural similarity. It is also interesting to note that cultural distance exerts more negative effects on socializing social media usage than on informational social media usage, which contributes to the scholarly efforts of unveiling a complete picture of how cultural distance influences social media usage in a cross-cultural context. And those research findings contribute well to our understandings pertaining to how to enhance users’ purchase intention on social commerce websites in a foreign environment with cultural novelty.

Thirdly, the research also extends the application of social learning theory to the social commerce research. Originated to serve psychological research, the social learning theory demonstrates its power to explicate individuals’ skill and knowledge acquisition in specific contexts (Tu, 2000; Yi and Davis, 2003; Hu et al., 2020). However, little research has ever employed this theoretical lens to illuminate individuals’ behaviors in social commerce research. The current research extended the theory to delineate users’ cognitive and emotional changes in fostering their purchase intention in culturally diversified social commerce environment. The research findings keep consistent with the proposal that social learning underpins social commerce application as a fundamental social psychology (Chen et al., 2017).

The current investigation also provides three insights pertaining to practical and managerial practices directed at promoting social commerce operations, particularly international business operations.

First, much attention should be paid to the significant enabling roles of social media usage in promoting users’ purchase intention on social commerce sites when international consumers are exposed to a culturally diversified business environment. Social media usage serves to positively influence users’ purchase intention on social commerce websites (Pradana and Ha, 2021). Measures encouraging potential consumers to use social media to surf the company’s social commerce websites or communicate with other consumers who are loyal to the company’s social commerce should be considered. Well-designed company’s social commerce websites with valuable and credible information posted by the company itself or shared by other consumers would facilitate potential consumers’ understandings about products and marketing strategies originated from cultures. Further, convenient communicative channels designed on social commerce websites serve better interactivity between community members and contribute to stickness of the company’s websites. Successful communications through the platform of social commerce websites contribute to the establishment of robust interpersonal relationships between consumers and offer avenues to spread products’ word-of-mouth (Hariguna et al., 2020). And this will accumulate interests and motivations of potential consumers and increase their purchase intention on social commerce websites.

Second, given that cultural intelligence plays the role of mediating the relationships between social media usage and users’ purchase intention on s-commerce sites, individuals with insufficient cultural intelligence tend to be less motivated to leverage social media to seek for necessary business information, fail to establish or maintain interpersonal relationships for intimate communications about business products, and finally lose their interests and purchase intention on social commerce websites. As such, any culture-related programs or activities including well-designed social commerce websites with necessary cultural knowledge to promote potential consumers’ knowledge about foreign products’ reflections of another country’s or region’s culture should be regarded as complementary and effective measures for appropriate marketing strategies. Those measures are supposed to develop cultural intelligence of targeted international consumers, thereby generating their purchase intention for online shopping behaviors. For international consumers, their capability to understand the culture nourishing the products and relevant business promotions seems to be crucial to determine their purchase decision. In this regard, measures promoting the cultural knowledge closely related to the products are strongly encouraged.

Finally, one important insight for practitioners to conduct successful international business operations in a foreign culture is that people with different cultural values should be addressed appropriately (Wankel, 2016). Thus cultural distance should be given full consideration during the process of product designing and promotion in a cross-cultural environment. Any marketing strategies promoting a company public image or culturally rooted products should be meticulously designed with serious consideration about the cultural dissimilarities between countries. Taken the event of famous Italian D and G Company as an example, which happened in November 2018, the company’s advertising activity for a fashion show organized in Shanghai, China was regarded as an insulting conflict against Chinese tradition and culture. This event gained rapid fermentation through social media and the company was confronted with its most serious marketing crisis in China. Thus being fully aware of potential risks brought by cultural distance is the very first step for practitioners to deliver products’ information on social media. Appropriate marketing approaches to convey and interpret culturally rooted products help to trigger a customer’s interest, intrinsic motivation, and cultural identification to develop their cultural intelligence and render their purchase intention on s-commerce sites. In addition, marketing strategies designed to seek cultural similarities or similar inherent values between target and home countries may attenuate the negative effects brought by cultural differences, which contributes to the user’s cultural intelligence and helps to generate more purchase intention.

In addition to the theoretical and practical implications, the current study makes relevant contributions to society as well. First of all, the study confirms the enabling roles of social media in social commerce. This confirms the mutually melt and integrative relationships between information technology advancement and business prosperity. Development of information technology changes the operation modes and enhances the success of every business. In the era of digitalization, business and technology should be further combined and integrated. Secondly, in the process of world’s economic globalization, businesses are supposed to confront increasing political, economic and cultural challenges. In response to those encounters, business development should consider global layout to avoid unexpected risks. Given the limited capacities of domestic markets, international approach could be one part of alternatives to increase business efficiency. The current study indicates that culture-relevant elements should be seriously considered for international business operations. Increasing cultural awareness and knowledge of international consumers is an effective conduit to enhance their purchase intention in cross-cultural social commerce. In this regard, cultural knowledge sharing between nations contributes to business success. Last but not the least, cultural distance, as indicated by previous research, has been identified as a dominating factor deterring interpersonal communications between individuals from different cultural backgrounds. Cultures demonstrate their effects from both positive and negative effects on human’s lives. On the one hand, prosperity in cultures finally constitutes one part of human’s glorious civilization. On the other hand, different cultures bring about obstacles to people’s communications and even social stability. In this regard, measures mitigating negative effects of cultural differences should be taken to avoid potential cultural conflicts and help people with different cultural identifications to enjoy other nations’ cultural glamor. And this finally vitalizes human’s development in every domain including business operations.

## Limitations and Future Avenues

Regardless of the above-mentioned important contributions, the study also has limitations that could be addressed as prospective research efforts. First, the selected sample for the current study could be problematic. Albeit we selected at random appropriate international students samples studying at Chinese universities, the majority of students participating in the survey were from Africa and Asia. The group of international students can be regarded as important international social commerce consumers, whereas there might be differences in other groups of international consumers. Further, he sampling areas limitation may narrow the generalization and application of research findings in other parts of the world. Therefore, researchers should be cautious to extend the research findings to other groups of international consumers. More empirical research should be replicated to re-examine the findings with samples from other countries.

Second, the research results indicate that cultural intelligence plays the role of mediating the relationships between social media usage and users’ purchase intention in culturally diversified social commerce environment. However, the current study only chose the key research construct in cross-cultural studies, cultural intelligence as the mediator aiming to unveil how social media usage exerts impact on consumers’ purchase intention. In addition, cultural intelligence was found only to partially mediate the relationships. Given the more complexity and uncertainty existing in culturally diversified social commerce business environment compared to a singular cultural background, more empirical research deems to be supplementary to further investigate any other mediators and situational factors to explicate how social media usage influences users’ purchase intention.

Finally, the research method to examine the proposed framework could be another problem. The adopted cross-sectional design only presents correlative, not causal relations between variables. For instance, the underlying mechanism of social media usage working on cultural intelligence could be reverse in such a way that individuals who possess sufficient cultural intelligence are supposed to rely on social media more heavily because they are more aware of the importance of social media in a foreign environment to help them develop personal capacities. Notwithstanding the conclusion reached with a longitudinal research design indicating that social media usage at first wave results in second-wave cultural intelligence (Hu et al., 2018), future empirical investigations are still necessary to offer a much clearer picture about antecedents determining users’ social commerce behaviors or intention in the future.

## Conclusion

Adopting social learning theory as the theoretical lens, the current research conducted an in-depth empirical study about cross-cultural social commerce. In particular, we adopted a multidisciplinary perspective by combining social commerce research and cross-cultural studies to investigate the underlying mechanism whereby social media usage exerts effects on users’ purchase intention via cultural intelligence, while also considering the attenuating effects of cultural distance. The current research serves as one of the first trials to call for researching social commerce with an interdisciplinary approach in the future efforts.

## Data Availability Statement

The raw data supporting the conclusions of this article will be made available by the authors, without undue reservation.

## Author Contributions

SH did the conceptualization, performed the methodology, and wrote the original draft. ZZ carried out the software, visualized and investigated the data, and wrote, reviewed, and edited the manuscript. Both authors contributed to the article and approved the submitted version.

## Conflict of Interest

The authors declare that the research was conducted in the absence of any commercial or financial relationships that could be construed as a potential conflict of interest.

## Publisher’s Note

All claims expressed in this article are solely those of the authors and do not necessarily represent those of their affiliated organizations, or those of the publisher, the editors and the reviewers. Any product that may be evaluated in this article, or claim that may be made by its manufacturer, is not guaranteed or endorsed by the publisher.

## Footnotes

1
www.wjx.cn

## References

[B1] Adiandari A. M. (2022). Financial performance innovation since digital technology entered Indonesian MSMEs. *Int. J. Appl. Inform. Manag.* 2 50–58.

[B2] Algharabat R. S. Rana N. P. (2021). Social commerce in emerging markets and its impact on online community engagement. *Inform. Syst. Front.* 23 1499–1520. 10.1007/s10796-020-10041-4

[B3] AlMazrouei H. Zacca R. (2021). Cultural intelligence as a predictor of expatriate managers turnover intention and creative self-efficacy. *Int. J. Organ. Anal.* 29 59–77. 10.1108/ijoa-10-2019-1904

[B4] Alnoor A. Al-Abrrow H. Al Halbusi H. Khaw K. W. Chew X. Al-Maatoq M. (2022). Uncovering the antecedents of trust in social commerce: an application of the non-linear artificial neural network approach. *Competitiveness Rev.* Epub ahead of print.

[B5] Ang S. Van Dyne L. (2015). *Handbook of Cultural Intelligence.* London: Routledge.

[B6] Ang S. Van Dyne L. Koh C. Ng K. Y. Templer K. J. Tay C. (2007). Cultural intelligence: its measurement and effects on cultural judgment and decision making, cultural adaptation, and task performance. *Manag. Organ. Rev.* 3 335–371. 10.1111/j.1740-8784.2007.00082.x

[B7] Aral S. Dellarocas C. Godes D. (2013). Social media and business transformation: a framework for research. *Inform. Syst. Res.* 24 3–13. 10.1287/isre.1120.0470 19642375

[B8] Bagozzi R. P. (1981). Evaluating structural equation models with unobservable variables and measurement error: a comment. *J. Mark. Res.* 18 375–381. 10.2307/3150979

[B9] Baltacı A. (2017). Relations between prejudice, cultural intelligence and level of entrepreneurship: a study of school principals. *Int. Electron. J. Elem. Educ.* 9 645–666.

[B10] Bandura A. (1978). Social learning theory of aggression. *J. Commun.* 28 12–29. 10.1111/j.1460-2466.1978.tb01621.x 690254

[B11] Black J. S. Mendenhall M. Oddou G. (1991). Toward a comprehensive model of international adjustment: an integration of multiple theoretical perspectives. *Acad. Manag. Rev.* 16 291–317. 10.5465/amr.1991.4278938

[B12] Bonebrake K. (2002). College students’ internet use, relationship formation, and personality correlates. *Cyberpsychol. Behav.* 5 551–557. 10.1089/109493102321018196 12556118

[B13] Bugshan H. Attar R. W. (2020). Social commerce information sharing and their impact on consumers. *Technol. Forecast. Soc. Chang.* 153:119875. 10.1016/j.techfore.2019.119875

[B14] Busalim A. H. Hussin A. R. C. (2016). Understanding social commerce: a systematic literature review and directions for further research. *Int. J. Inform. Manag.* 36 1075–1088. 10.1016/j.ijinfomgt.2016.06.005

[B15] Chavis A. M. (2011). Social learning theory and behavioral therapy: considering human behaviors within the social and cultural context of individuals and families. *Soc. Work Public Health* 26 471–481. 10.1080/19371918.2011.591629 21902482

[B16] Chawla N. Kumar B. (2021). E-commerce and consumer protection in india: the emerging trend. *J. Bus. Ethics* 1, 1–24. 10.1007/s10551-021-04884-3 34257470 PMC8267237

[B17] Chen A. Lu Y. Wang B. (2017). Customers’ purchase decision-making process in social commerce: a social learning perspective. *Int. J. Inform. Manag.* 37 627–638. 10.1016/j.ijinfomgt.2017.05.001

[B18] Chen G. Kirkman B. L. Kim K. Farh C. I. C. Tangirala S. (2010). When does cross-cultural motivation enhance expatriate effectiveness? a multilevel investigation of the moderating roles of subsidiary support and cultural distance. *Acad. Manag. J.* 53 1110–1130. 10.5465/amj.2010.54533217

[B19] Chen J. Shen X. L. (2015). Consumers’ decisions in social commerce context: an empirical investigation. *Decis. Support Syst.* 79 55–64. 10.1016/j.dss.2015.07.012

[B20] Chen N. Chao M. C. Xie H. Tjosvold D. (2018). Transforming cross-cultural conflict into collaboration: the integration of western and eastern values. *Cross Cult. Strateg. Manag.* 25 70–95. 10.1108/ccsm-10-2016-0187

[B21] Chen X. P. Liu D. Portnoy R. (2012). A multilevel investigation of motivational cultural intelligence, organizational diversity climate, and cultural sales: evidence from US real estate firms. *J. Appl. Psychol.* 97 93–106. 10.1037/a0024697 21806296

[B22] Chen X. P. Peng S. (2008). Guanxi dynamics: shifts in the closeness of ties between Chinese coworkers. *Manag. Organ. Rev.* 4 63–80. 10.1111/j.1740-8784.2007.00078.x

[B23] Curty R. G. Zhang P. (2013). Website features that gave rise to social commerce: a historical analysis. *Electron. Commer. Res. Appl.* 12 260–279. 10.1016/j.elerap.2013.04.001

[B24] Deng Z. Wang Z. (2016). Early-mover advantages at cross-border business-to-business e-commerce portals. *J. Bus. Res.* 69 6002–6011. 10.1016/j.jbusres.2016.05.015

[B25] Earley P. C. Ang S. (2003). *Cultural Intelligence: Individual Interactions across Cultures.* Stanford, CA: Stanford University Press.

[B26] Ellison N. B. Steinfield C. Lampe C. (2007). The benefits of Facebook “friends:” social capital and college students’ use of online social network sites. *J. Comput. Med. Commun.* 12 1143–1168. 10.1111/j.1083-6101.2007.00367.x

[B27] English A. S. Zhang Y. B. Tong R. (2021). Social support and cultural distance: sojourners’ experience in China. *Int. J. Intercult. Relat.* 80 349–358. 10.1016/j.ijintrel.2020.10.006

[B28] Fornell C. Larcker D. F. (1981). Evaluating structural equation models with unobservable variables and measurement error. *J. Mark. Res.* 18 39–50. 10.2307/3151312

[B29] Friedman T. L. (2006). *The World is Flat: A Brief History of the Twenty-First Century (Rev. ed.).* New York, NY: Farrar, Straus and Giroux.

[B30] Friedrich T. Schlauderer S. Overhage S. (2019). The impact of social commerce feature richness on website stickiness through cognitive and affective factors: an experimental study. *Electron. Commer. Res. Appl.* 36:100861. 10.1016/j.elerap.2019.100861

[B31] Frijns B. Garel A. (2021). The effect of cultural distance between an analyst and a CEO on analysts’ earnings forecast performance. *Econ. Lett.* 205:109957. 10.1016/j.econlet.2021.109957

[B32] Froese F. J. Peltokorpi V. (2011). Cultural distance and expatriate job satisfaction. *Int. J. Intercult. Relat.* 35 49–60. 10.1016/j.ijintrel.2010.10.002

[B33] Fu J.-R. Lu I. W. Chen J. H. F. Farn C.-K. (2020). Investigating consumers’ online social shopping intention: an information processing perspective. *Int. J. Inform. Manag.* 54:102189. 10.1016/j.ijinfomgt.2020.102189

[B34] Galchenko I. van de Vijver F. J. R. (2007). The role of perceived cultural distance in the acculturation of exchange students in Russia. *Int. J. Intercult. Relat.* 31 181–197. 10.1016/j.ijintrel.2006.03.004

[B35] Gassmann O. Zeschky M. Wolff T. Stahl M. (2010). Crossing the industry-line: breakthrough innovation through cross-industry alliances with ‘non-suppliers’. *Long Range Plann.* 43 639–654. 10.1016/j.lrp.2010.06.003

[B36] Ghahtarani A. Sheikhmohammady M. Rostami M. (2020). The impact of social capital and social interaction on customers’ purchase intention, considering knowledge sharing in social commerce context. *J. Innov. Knowl.* 5 190–198.

[B37] Glass C. R. Westmont C. M. (2014). Comparative effects of belongingness on the academic success and cross-cultural interactions of domestic and international students. *Int. J. Intercult. Relat.* 39 152–163.

[B38] Guan L. Liang H. Zhu J. J. H. (2022). Predicting reposting latency of news content in social media: a focus on issue attention, temporal usage pattern, and information redundancy. *Comput. Hum. Behav.* 127:107080. 10.1016/j.chb.2021.107080

[B39] Guang X. Charoensukmongkol P. (2022). The effects of cultural intelligence on leadership performance among Chinese expatriates working in Thailand. *Asian Bus. Manag.* 21 106–128. 10.1057/s41291-020-00112-4

[B40] Guillaume Y. R. F. Knippenberg D. V. Brodbeck F. C. (2014). Nothing succeeds like moderation: a social self-regulation perspective on cultural dissimilarity and performance. *Acad. Manag. J.* 57 1284–1308. 10.5465/amj.2013.0046

[B41] Gutama D. H. Umami I. Saputro P. H. (2021). Analysis of the effect of website sales quality on purchasing decisions on e-commerce websites. *Int. J. Inform. Inform. Syst.* 4 71–81. 10.47738/ijiis.v4i1.79

[B42] Hair J. F. Jr. Black W. C. Babin B. J. Anderson R. E. (2020). *Multivariate Data Analysis*, 8th Edn. Upper Saddle River, NJ: Prentice Hall.

[B43] Hajli N. (2015). Social commerce constructs and consumer’s intention to buy. *Int. J. Inform. Manag.* 35 183–191. 10.1016/j.ijinfomgt.2014.12.005

[B44] Hariguna T. Rachmawati V. (2019). Community opinion sentiment analysis on social media using naive bayes algorithm methods. *Int. J. Inform. Inform. Syst.* 2 33–38. 10.47738/ijiis.v2i1.11

[B45] Hariguna T. Sukmana H. T. Kim J. I. (2020). Survey opinion using sentiment analysis. *J. Appl. Data Sci.* 1 35–40. 10.47738/jads.v1i1.10

[B46] Hoppner J. J. Griffith D. A. White R. C. (2015). Reciprocity in relationship marketing: a cross-cultural examination of the effects of equivalence and immediacy on relationship quality and satisfaction with performance. *J. Int. Mark.* 23 64–83. 10.1509/jim.15.0018 11670861

[B47] Hossain M. S. Rahman M. F. Zhou X. (2021). Impact of customers’ interpersonal interactions in social commerce on customer relationship management performance. *J. Contemp. Mark. Sci.* 4 161–181. 10.1108/jcmars-12-2020-0050

[B48] Hu S. Gu J. Liu H. Huang Q. (2017). The moderating role of social media usage in the relationship among multicultural experiences, cultural intelligence, and individual creativity. *Inform. Technol. People* 30 265–281. 10.1108/itp-04-2016-0099

[B49] Hu S. Hu L. Wang G. (2021a). Moderating role of addiction to social media usage in managing cultural intelligence and cultural identity change. *Inform. Technol. People* 34 704–730. 10.1108/itp-10-2019-0518

[B50] Hu S. Hu L. Wu J. Wang G. (2021b). Social media usage and international expatriate’s creativity: an empirical research in cross-cultural context. *Hum. Manag. Syst.* 40 197–209. 10.3233/hsm-200965

[B51] Hu S. Liu H. Gu J. (2018). What role does self-efficacy play in developing cultural intelligence from social media usage? *Electron. Commer. Res. Appl.* 28 172–180. 10.1016/j.elerap.2018.01.009

[B52] Hu S. Liu H. Zhang S. Wang G. (2020). Proactive personality and cross-cultural adjustment: roles of social media usage and cultural intelligence. *Int. J. Intercult. Relat.* 74 42–57. 10.1016/j.ijintrel.2019.10.002

[B53] Huang Z. Benyoucef M. (2013). From e-commerce to social commerce: a close look at design features. *Electron. Commer. Res. Appl.* 12 246–259. 10.1016/j.elerap.2012.12.003

[B54] Huang Z. Benyoucef M. (2015). User preferences of social features on social commerce websites: an empirical study. *Technol. Forecast. Soc. Chang.* 95 57–72. 10.1016/j.techfore.2014.03.005

[B55] Hughes D. J. Rowe M. Batey M. Andrew L. (2012). A tale of two sites: Twitter vs. Facebook and the personality predictors of social media usage. *Comput. Hum. Behav.* 28 561–569. 10.1016/j.chb.2011.11.001

[B56] Hur K. Kim T. T. Karatepe O. M. Lee G. (2017). An exploration of the factors influencing social media continuance usage and information sharing intentions among Korean travelers. *Tour. Manag.* 63 170–178. 10.1016/j.tourman.2017.06.013

[B57] Hwang I. J. Lee B. G. Kim K. Y. (2014). Information asymmetry, social networking site word of mouth, and mobility effects on social commerce in Korea. *Cyberpsychol. Behav. Soc. Netw.* 17 117–124. 10.1089/cyber.2012.0566 24355038

[B58] Illeris K. (2003). Towards a contemporary and comprehensive theory of learning. *Int. J. Lifelong Educa.* 22 396–406. 10.1080/02601370304837

[B59] Imai L. Gelfand M. J. (2010). The culturally intelligent negotiator: the impact of cultural intelligence (CQ) on negotiation sequences and outcomes. *Organ. Behav. Hum. Decis. Process.* 112 83–98. 10.1016/j.obhdp.2010.02.001

[B60] Jami Pour M. Hosseinzadeh M. Mansouri N. S. (2022). Challenges of customer experience management in social commerce: an application of social network analysis. *Internet Res.* 32 241–272. 10.1108/intr-01-2021-0076

[B61] Jen L. Lin Y. (2021). A brief overview of the accuracy of classification algorithms for data prediction in machine learning applications. *J. Appl. Data Sci.* 2 84–92. 10.47738/jads.v2i3.38

[B62] Jin X. Chen X. Zhou Z. (2022). The impact of cover image authenticity and aesthetics on users’ product-knowing and content-reading willingness in social shopping community. *Int. J. Inform. Manag.* 62:102428. 10.1016/j.ijinfomgt.2021.102428

[B63] Karatas K. Arpaci I. (2021). The mediating role of tolerance in the relationship between cultural intelligence and xenophobia. *Asia Pac. Educ. Rev.* 22 119–127. 10.1007/s12564-021-09675-z

[B64] Kauffman R. J. Kim K. Lee S. Y. Hoang A. P. Ren J. (2017). Combining machine based and econometrics methods for policy analytics insights. *Electron. Commer. Res. Appl.* 25 115–140. 10.1016/j.elerap.2017.04.004

[B65] Khaola P. P. Musiiwa D. Rambe P. (2022). The influence of social media usage and student citizenship behaviour on academic performance. *Int. J. Manag. Educ.* 20:100625. 10.1002/ijop.12200 26242614

[B66] Kim S. Park H. (2013). Effects of various characteristics of social commerce (s-commerce) on consumers’ trust and trust performance. *Int. J. Inform. Manag.* 33 318–332. 10.1016/j.ijinfomgt.2012.11.006

[B67] Kim Y. J. Dyne L. V. (2012). Cultural intelligence and international leadership potential: the importance of contact for members of the majority. *Appl. Psychol. Int. Rev.* 61 272–294. 10.1111/j.1464-0597.2011.00468.x

[B68] Ko H. C. (2018). Social desire or commercial desire? The factors driving social sharing and shopping intentions on social commerce platforms. *Electron. Commer. Res. Appl.* 28 1–15. 10.1016/j.elerap.2017.12.011

[B69] Kotabe M. Srinivasan S. S. Aulakh P. S. (2002). Multinationality and firm performance: the moderating role of RandD and marketing capabilities. *J. Int. Bus. Stud.* 33 79–97. 10.1057/palgrave.jibs.8491006

[B70] Lankton N. K. McKnight D. H. Wright R. T. Thatcher J. B. (2016). Research note—using expectation disconfirmation theory and polynomial modeling to understand trust in technology. *Inform. Syst. Res.* 27 197–213. 10.1287/isre.2015.0611 19642375

[B71] LaRose R. (2009). “Social cognitive theories of media selection,” in *Media Choice: A Theoretical and Empirical Overview*, ed. Hartmann T. (New York, NY: Routledge), 10–31.

[B72] Lee L. Y. Sukoco B. M. (2010). The effffects of cultural intelligence on expatriate performance: the moderating effffects of international experience. *Int. J. Hum. Resour. Manag.* 21 963–981. 10.1080/09585191003783397

[B73] Leong L.-Y. Hew T.-S. Ooi K.-B. Chong A. Y.-L. (2020). Predicting the antecedents of trust in social commerce – a hybrid structural equation modeling with neural network approach. *J. Bus. Res.* 110 24–40. 10.1016/j.jbusres.2019.11.056

[B74] Li C. Tsai W. H. S. (2015). Social media usage and acculturation: a test with Hispanics in the U.S. *Comput. Hum. Behav.* 45 204–212. 10.1016/j.chb.2014.12.018

[B75] Liang T. P. Turban E. (2011). Introduction to the special issue on social commerce: a research framework for social commerce. *Int. J. Electron. Commer.* 16 5–13. 10.2753/jec1086-4415160201

[B76] Lickel B. Hamilton D. L. Wieczorkowska G. Lewis A. Sherman S. J. Uhles A. N. (2000). Varieties of groups and the perception of group entitativity. *J. Pers. Soc. Psychol.* 78 223–246. 10.1037//0022-3514.78.2.223 10707331

[B77] Lin C. S. Kuo F. Y. Hung C. Y. (2021). Exploring social media use and civic engagement on the discussion of antinuclear issue. *Int. J. Appl. Inform. Manag.* 2 90–96. 10.47738/ijaim.v2i2.31

[B78] Lin X. Li Y. Wang X. (2017). Social commerce research: definition, research themes and the trends. *Int. J. Inform. Manag.* 37 190–201. 10.1016/j.ijinfomgt.2016.06.006

[B79] Liu A. Lu C. Wang Z. (2021). Does cultural distance hinder exports?: a comparative study of China and the United States. *Econ. Model.* 105 105668. 10.1016/j.econmod.2021.105668

[B80] Lorenzo O. Kawalek P. Ramdani B. (2012). Enterprise applications diffusion within organizations: a social learning perspective. *Inform. Manag.* 49 47–57. 10.1016/j.im.2011.10.005

[B81] Lu B. Fan W. Zhou M. (2016). Social presence, trust, and social commerce purchase intention: an empirical research. *Comput. Hum. Behav.* 56 225–237. 10.1016/j.chb.2015.11.057

[B82] Ma Z. (2010). The SINS in business negotiations: explore the cross-cultural differences in business ethics between Canada and China. *J. Bus. Ethics* 91 123–135. 10.1007/s10551-010-0571-5

[B83] MacNab B. R. Worthley R. (2012). Individual characteristics as predictors of cultural intelligence development: the relevance of self-effiffifficacy. *Int. J. Intercult. Relat.* 36 62–71. 10.1016/j.ijintrel.2010.12.001

[B84] Matsuda T. (2022). Research on new regional creation business model utilizing social network and crowdfunding. *Int. J. Appl. Inform. Manag.* 2 1–12. 10.47738/ijaim.v2i1.22

[B85] McCort D. J. Malhotra N. K. (1993). Culture and consumer behavior: toward an understanding of cross-cultural consumer behavior in international marketing. *J. Int. Consum. Mark.* 6 91–127. 10.1300/j046v06n02_07

[B86] Mendenhall M. Oddou G. (1985). The dimensions of expatriate acculturation: a review. *Acad. Manag. Rev.* 10 39–47. 10.5465/amr.1985.4277340

[B87] Mikalef P. Giannakos M. N. Pappas I. O. (2017). Designing social commerce platforms based on consumers’ intentions. *Behav. Inform. Technol.* 36 1308–1327. 10.1080/0144929x.2017.1386713

[B88] Ministry of Education of People’s Republic of China (2018). *Statistics of International Students Studying in China 2018.* Available online at: http://www.moe.gov.cn/jyb_xwfb/gzdt_gzdt/s5987/201904/t20190412_377692.html (accessed April 12, 2019).

[B89] Mohammed E. Qhal A. (2020). Knowledge management strategy by means of virtualization in covid-19. *J. Appl. Data Sci.* 1 41–53. 10.7759/cureus.15699 34277286 PMC8285254

[B90] Nacar R. Ozdemir K. (2022). “From commerce to E-commerce and social commerce: how global? How local?,” in *Industry 4.0 and Global Businesses*, ed. Yakut E. (Bingley: Emerald Publishing Limited), 95–109. 10.1108/978-1-80117-326-120211007

[B91] Ng C. S. P. (2013). Intention to purchase on social commerce websites across cultures: a cross-regional study. *Inform. Manag.* 50 609–620. 10.1016/j.im.2013.08.002

[B92] Osatuyi B. Qin H. Osatuyi T. Turel O. (2020). When it comes to satisfaction …It depends: an empirical examination of social commerce users. *Comput. Hum. Behav.* 111:106413. 10.1016/j.chb.2020.106413

[B93] Paireekreng W. Osathanukroh J. Supasak C. (2019). A study of influence factors for advertising on messaging applications towards mobile buyer’s decision making. *Int. J. Inform. Inform. Syst.* 2 82–90.

[B94] Pidduck R. J. Shaffer M. A. Zhang Y. Cheung S. S. Y. Yunlu D. G. (2022). Cultural intelligence: an identity lens on the influence of cross-cultural experience. *J. Int. Manag.* 28:100928. 10.1016/j.intman.2022.100928

[B95] Podsakoff P. M. Mackenzie S. B. Podsakoff N. P. (2012). Sources of method bias in social science research and recommendations on how to control it. *Annu. Rev. Psychol.* 63 539–569. 10.1146/annurev-psych-120710-100452 21838546

[B96] Prabowo N. A. Pujiarto B. Wijaya F. S. Gita L. Alfandy D. (2021). Social network analysis for user interaction analysis on social media regarding e-commerce business. *Int. J. Inform. Inform. Syst.* 4 95–102. 10.47738/ijiis.v4i2.106

[B97] Pradana M. G. Ha H. T. (2021). Maximizing strategy improvement in mall customer segmentation using K-means clustering. *J. Appl. Data Sci.* 2 19–25.

[B98] Presbitero A. (2018). Extraversion, openness to experience, and global career intention: the mediating role of cultural intelligence. *J. Employ. Couns.* 55 104–114. 10.1002/joec.12090

[B99] Priyo J. S. Mohamad B. Adetunji R. R. (2019). An examination of the effects of service quality and customer satisfaction on customer loyalty in the hotel industry. *Int. J. Supply Chain Manag.* 8 653–663.

[B100] Rakhmansyah M. Wahyuningsih T. Srenggini A. D. Gunawan I. K. (2022). Small and medium enterprises (SMEs) with SWOT analysis method. *Int. J. Appl. Inform. Manag.* 2 47–54. 10.47738/ijaim.v2i3.37

[B101] Schumann J. H. Wangenheim F. V. Stringfellow A. Yang Z. Blazevic V. Praxmarer S. (2010). Cross-cultural differences in the effect of received word-of-mouth referral in relational service exchange. *J. Int. Mark.* 18 62–80. 10.1509/jimk.18.3.62 11670861

[B102] Shenkar O. (2001). Cultural distance revisited: towards a more rigorous conceptualization and measurement of cultural differences. *J. Int. Bus. Stud.* 32 1–17. 10.1057/jibs.2011.40

[B103] Shin D. H. (2013). User experience in social commerce: in friends we trust. *Behav. Inform. Technol.* 32 52–67. 10.1080/0144929x.2012.692167

[B104] Silbiger A. Barnes B. R. Berger R. Renwick D. W. S. (2021). The role of regulatory focus and its influence on the cultural distance – adjustment relationship for expatriate managers. *J. Bus. Res.* 122 398–410. 10.1016/j.jbusres.2020.09.021

[B105] Soleimani M. (2021). Buyers’ trust and mistrust in e-commerce platforms: a synthesizing literature review. *Inform. Syst. Ebus. Manag.* 10.1007/s10257-021-00545-0 [Epub ahead of print].

[B106] Stewart K. J. (2003). Trust transfer on the world wide web. *Organ. Sci.* 14 5–17. 10.1287/orsc.14.1.5.12810 19642375

[B107] Stewart S. M. Bing M. N. (2009). In the eyes of the beholder: a non-self-report measure of workplace deviance. *J. Appl. Psychol.* 94 207–215. 10.1037/a0012605 19186905

[B108] Stoermer S. Davies S. Froese F. J. (2021). The influence of expatriate cultural intelligence on organizational embeddedness and knowledge sharing: the moderating effects of host country context. *J. Int. Bus. Stud.* 52 432–453. 10.1057/s41267-020-00349-3

[B109] Sun Y. Shao X. Li X. Guo Y. Nie K. (2019). How live streaming influences purchase intentions in social commerce: an IT affordance perspective. *Electron. Commer. Res. Appl.* 37:100886. 10.1016/j.elerap.2019.100886

[B110] Tang L. (2017). Mine your customers or mine your business: the moderating role of culture in online word-of-mouth reviews. *J. Int. Mark.* 25 88–110. 10.1509/jim.16.0030 11670861

[B111] Tett R. P. Burnett D. D. (2003). A personality trait-based interactionist model of job performance. *J. Appl. Psychol.* 88 500–517. 10.1037/0021-9010.88.3.500 12814298

[B112] Triandis H. C. (2006). Cultural intelligence in organizations. *Group Organ. Manag.* 31 20–26. 10.1177/1059601105275253

[B113] Tu C. H. (2000). On-line learning migration: from social learning theory to social presence theory in a CMC environment. *J. Netw. Comput. Appl.* 23 27–37. 10.1006/jnca.1999.0099

[B114] Van Heel B. Lukic V. Leeuwis E. (2011). *Cross-Border E-Commerce Makes the World Flatter.* Boston, MA: Boston Consulting Group.

[B115] Wang C. Yang Y. Y. Chiang M. H. (2021). Understanding users’ attitude to social endorsement advertising of embarrassing product. *Int. J. Appl. Inform. Manag.* 1 6–22.

[B116] Wang C. Yang Y. Y. Lin M. (2019). The influence of the privacy concern and social advertising type on the attitude and behavior. *Int. J. Inform. Inform. Syst.* 2 75–81. 10.2196/15455 31670698 PMC6914244

[B117] Wang E. S. T. (2010). Internet usage purposes and gender differences in the effects of perceived utilitarian and hedonic value. *Cyberpsychol. Behav. Soc. Netw.* 13 179–183. 10.1089/cyber.2009.0200 20528275

[B118] Wang X. Lin X. Spencer M. K. (2019). Exploring the effects of extrinsic motivation on consumer behaviors in social commerce: revealing consumers’ perceptions of social commerce benefits. *Int. J. Inform. Manag.* 45 163–175. 10.1016/j.ijinfomgt.2018.11.010

[B119] Wang Y. Herrando C. (2019). Does privacy assurance on social commerce sites matter to millennials? *Int. J. Inform. Manag.* 44 164–177. 10.1016/j.ijinfomgt.2018.10.016

[B120] Wankel C. (2016). Developing cross-cultural managerial skills through social media. *J. Organ. Chang. Manag.* 29 116–124. 10.1108/jocm-11-2015-0225

[B121] Ward C. Wilson J. Ronald F. (2011). Assessing the predictive validity of cultural intelligence over time. *Pers. Individ. Differ.* 51 138–142. 10.1016/j.paid.2011.03.032

[B122] Weller K. (2016). Trying to understand social media users and usage: the forgotten features of social media platforms. *Online Inform. Rev.* 40 256–264. 10.1108/oir-09-2015-0299

[B123] Wigand R. T. Benjamin R. I. Birkland J. L. H. (2008). “Web 2.0 and beyond: implications for electronic commerce,” in *Proceedings of the 10th International Conference on Electronic Commerce, Innsbruck, Austria* (New York, NY: ACM Press).

[B124] Yadav M. S. de Valck K. Hennig-Thurau T. Hoffman D. L. Spann M. (2013). Social commerce: a contingency framework for assessing marketing potential. *J. Interact. Mark.* 27 311–323. 10.1016/j.intmar.2013.09.001

[B125] Yazdanshenas M. (2021). Core self-evaluations and project managers’ competencies: the moderating role of cultural intelligence. *J. Manag. Dev.* 40 542–573. 10.1108/jmd-01-2021-0031

[B126] Yeo G. B. Neal A. (2004). A multilevel analysis of effort, practice and performance: effects of ability, conscientiousness and goal orientation. *J. Appl. Psychol.* 89 231–247. 10.1037/0021-9010.89.2.231 15065972

[B127] Yi M. Y. Davis F. D. (2003). Developing and validating an observational learning model of computer software training and skill acquisition. *Inform. Syst. Res.* 14 146–169. 10.1287/isre.14.2.146.16016 19642375

[B128] Zhang J. Goodson P. (2011). Acculturation and psychosocial adjustment of Chinese international students: examining mediation and moderation effects. *Int. J. Intercult. Relat.* 35 614–627. 10.1016/j.ijintrel.2010.11.004

[B129] Zhang K. Z. K. Benyoucef M. (2016). Consumer behavior in social commerce: a literature review. *Decis. Support Syst.* 86 95–108.

[B130] Zheng X. Men J. Xiang L. Yang F. (2020). Role of technology attraction and parasocial interaction in social shopping websites. *Int. J. Inform. Manag.* 51:102043. 10.1016/j.ijinfomgt.2019.102043

[B131] Zhou L. Zhang P. Zimmermann H. D. (2013). Social commerce research: an integrated view. *Electron. Commer. Res. Appl.* 12 61–68. 10.1016/j.elerap.2013.02.003

